# MOSH Syndrome (Male Obesity Secondary Hypogonadism): Clinical Assessment and Possible Therapeutic Approaches

**DOI:** 10.3390/nu10040474

**Published:** 2018-04-12

**Authors:** Antonino De Lorenzo, Annalisa Noce, Eleonora Moriconi, Tiziana Rampello, Giulia Marrone, Nicola Di Daniele, Valentina Rovella

**Affiliations:** 1 Section of Clinical Nutrition and Nutrigenomic, Department of Biomedicine and Prevention, University of Rome “Tor Vergata”, via Montpellier 1, 00133 Rome, Italy; delorenzo@uniroma2.it (A.D.L.); tiazianarampello1@gmail.com (T.R.); 2 Casa di Cura Madonna dello Scoglio, Traversa Mola, 88836 Cotronei, Italy; 3 Department of Medicine, Hypertension and Nephrology Unit, University Hospital “Tor Vergata”, viale Oxford 81, 00133 Rome, Italy; giul.marr@gmail.com (G.M.); valerovix@yahoo.it (V.R.); 4 Nutrition Service, “Nuova Clinica Annunziatella”, via Meropia 124, 00147 Rome, Italy; eleonoramoriconi87@gmail.com; 5 Specialization School of Food Science, University of Rome “Tor Vergata”, via Montpellier 1, 00133 Rome, Italy; 6 Unit of Endocrinology and Metabolic Diseases, Department of Systems Medicine, CTO “A. Alesini” Hospital, University of Rome “Tor Vergata”, via Montpellier 1, 00133 Rome, Italy; 7 School of Applied Medical-Surgical Sciences, University of Rome “Tor Vergata”, via Montpellier 1, 00133 Rome, Italy

**Keywords:** MOSH syndrome, lifestyle change, food addiction, Aromatase activity, Testosterone/17-Beta Estradiol Ratio

## Abstract

Male obesity secondary hypogonadism (MOSH) impairs fertility, sexual function, bone mineralization, fat metabolism, cognitive function, deteriorates muscle mass and alters body composition. The aim of this pilot study was to evaluate the effect of dietary intervention and physical activity on the MOSH patient’s hormonal profile after a 10% weight loss compared to baseline. Fourteen male patients were enrolled. Hormonal, lipid, glycemic profiles and body composition were determined at baseline and after a 10% weight loss. Aging Male Symptoms Scale (AMS) and Yale Food Addiction Scale (YFAS) were administered to patients in order to investigate hypogonadal symptoms and food addiction. Compared to baseline, a significant increase of Total Testosterone (TT) (300.2 ± 79.5 ng/dL vs. 408.3 ± 125.9 ng/dL, *p* = 0.002, 95% CI 26.8; 167.7) and a reduction of 17-Beta Estradiol level (48.3 ± 14.9 pg/mL vs. 39.2 ± 15.2 pg/mL, *p* = 0.049, 95% CI 3.1; 0.0) were observed. Total Fat Mass (FM) percentage, android and gynoid fat mass percentage (39.2 ± 6.4% vs. 36.2 ± 5.8%, *p* = 0.0001, 95% CI 22.5; 62.3; 51.5 ± 6.8% vs. 47.6 ± 6.8%, *p* = 0.001, 95% CI 0.6; 1.8, vs. 39.2 ± 6.2% vs. 36.5 ± 6.3% *p* = 0.0001, 95% CI 0.9; 2.0 respectively) were significantly decreased after nutritional intervention. In addition, total Fat Free Mass (FFM) in kg was significantly reduced after 10% weight loss (62.3 ± 2.8 kg vs. 60.3 ± 7.7 kg, *p* = 0.002, 95% CI 45.0; 93.0). Lifestyle changes, specifically dietotherapy and physical activity, induce positive effects on hypogonadism due to obesity.

## 1. Introduction

One of the major public health problems in the West is obesity. The toll on quality of life is linked to several metabolic disorders caused by excessive adipose tissue. Obesity causes a chronic low-grade inflammatory state, together with an overflow of free fatty acids into the blood stream and their subsequent ectopic deposition in vital organs [1,2]. According to non-communicable disease Risk Factor Collaboration (NCD-RisC) in 2014, about 266 million men and 375 million women were obese worldwide [3]. In the past decades, a dramatic increase in its prevalence has also been observed in children [4].

Hypogonadism is defined as a clinical condition characterized by altered gonadal function and androgen deficiency [5]. Male hypogonadism is more prevalent in middle-aged men (prevalence varies from 2.1 to 12.8%) [6]. However, recent studies showed that prevalence of hypogonadism has increased over the last 10 years, and this condition is actually underestimated and underdiagnosed. Sexually transmitted diseases, endocrine disruptors and obesity represent potential emerging risk factors for male infertility [7,8,9,10]. In obese men, hypogonadism is frequently a co-factor and it is strictly related to excess body fat and high plasma levels of leptin [11]. This pathological condition impairs fertility, sexual function, bone mineralization, fat metabolism, cognitive function, deteriorates muscle mass and alters body composition [12].

Several studies demonstrated that a modest weight reduction (approximately 10%) was ableto increase longevity and prevent the onset of chronic non-communicable diseases in obesepeople [13,14]. The expansion of adipose tissue is the basis of obesity and its comorbidities. In particular, male obesity is frequently associated with low Total Testosterone (TT) levels. Hypogonadism is often underdiagnosed despite its great impact on quality of life. It can cause erectile dysfunction, gynecomastia, low bone mineral density, low libido and sarcopenia. Furthermore, low testosterone levels exacerbate male obesity, facilitating adipose tissue deposition in visceral sites. This crosstalk between adipose tissue and testis creates a vicious cycle with deterioration in health status and life quality.

Male obesity secondary hypogonadism (MOSH) pathogenesis seems to be multifactorial and its three major risk factors are hyperestrogenism, metabolic endotoxemia and hyperleptinemia.

The first risk factor is constituted by adipose tissue expansion correlated to weight gain, which is linked to an overexpression of the enzyme aromatase. This enzyme converts Testosterone into Estradiol (Testosterone-Estradiol shunt) [15]. The hyperestrogenism decreases lutein hormone (LH) pituitary secretion through a negative feedback action that impairs the synthesis and production of testosterone from Leydig cells [16].

The second risk factor is metabolic endotoxemia. Tremellen et al., in GELDING theory (Gut Endotoxin Leading to a Decline In Gonadal function), hypothesized that hypercaloric and hyperlipidic diet causes the breakdown of the normal leaky gut, thus facilitating the passage of bacterial endotoxin from gut lumen into the blood stream (metabolic endotoxemia). Testosterone has an immunosuppressive action, resulting in a reduced ability of the individual to fight infections. Therefore, according to the GELDING theory, an evolution of the male reproductive axis skewed towards a reduced production of Testosterone in the case of prolonged exposure to bacterial endotoxins, would achieve a consequent decrease of its immunosuppressive action. This innovative theory associates obesity, metabolic endotoxemia and altered testicular function [17].

Several animal studies suggest that bacterial endotoxin (Lipopolysaccharides-LPS) could be able to reduce testicular function by binding toll-like receptor 4 (TLR4) on Leydig cells, stimulating the production of inflammatory cytokines [18,19].

Finally, the third risk factor is hyperleptinemia. Several studies have demonstrated that an enhanced level of leptin, as observed in obese men, strongly inhibits human chorionic gonadotropin (hCG)-stimulated androstenedione. This evidence seems to relate to the Fat Mass percentage (FM%) and leptin levels. Caprio et al. first highlighted the expression of leptin receptors (OB-R) in murine and human Leydig cells [11].

Obesity is often characterized by a compulsive intake of food and the inability not to eat rather than the desire to do so. These symptoms are overlapping to those described in Diagnostic and Statistical Manual of Mental Disorders-IV (DSM-IV) for substances and drug dependence. For this reason, obesity may be considered a “food addiction” [20]. Several studies suggest that the endogenous cannabinoid and opioid systems are the main circuits of response to the rewarding value of food [21]. In our pilot study, conducted on obese patients, we considered appropriate to evaluate the possible presence of food addiction.

We hereby refer to our study as a pilot given that the enrolled patients are exiguous in number. The aim of this study was to evaluate the hormonal profile of obese adults before and after nutritional intervention and physical activity (PA), aimed at achieving a weight loss of 10%. Particularly focusing on the activity of the enzyme aromatase determined by the Total Testosterone/17-Beta Estradiol ratio. Moreover, food addiction and hypogonadal signs and/or symptoms were assessed through the Aging Male Symptoms Scale (AMS) [22] and Yale Food Addiction Scale (YFAS) [23].

## 2. Materials and Methods

Twenty patients were screened from January–September 2016, amongst the centers of the Clinical Nutrition Service of “Tor Vergata” University of Rome (Italy) and in the Unit of Endocrinology and Metabolic Diseases, Department of Systems Medicine, “A. Alesini” Hospital of Rome (Italy). Inclusion criteria were: age 18–65 years, FM% > 30% estimated by DXA (dual-energy X-ray absorptiometry) examination, signs and symptoms of hypogonadism and TT < 12.1 nmol/L (349 ng/dL). According to guidelines on male hypogonadism, the cut-off is 12.1 nmol/L. It was selected because it allows to discern whether the TT values are normal or associated with deficiency. In this range of values (12.1–8.0 nmol/L), it is necessary in order to make a diagnosis of hypogonadism to evaluate the presence of three sexual symptoms. Namely, decreased sexual thoughts, weakened morning erections and erectile dysfunction [24,25].

Exclusion criteria were: major psychiatric diseases, cancers, infections and active autoimmune diseases. Finally, 14 Caucasian, male patients with secondary hypogonadism were recruited.

The protocol was written according to the ethical guidelines of Helsinki Declaration and was approved by the ‘Tor Vergata’ University Medical Ethical Committee. All patients enrolled in the study have provided signed consent, before being enrolled in the study.

Body composition and laboratory parameters were determined at baseline and after a 10% weight loss obtained by nutritional intervention.

### 2.1. Analysis of Blood Samples

Early morning blood samples were taken from each patient at baseline and after a 10% weight loss in order to characterize hormonal (TT, LH, sex hormone binding globulin-SHBG, albumin, prolactin, 17-Beta Estradiol-E2, 25-OH vitamin D), lipidemic (total cholesterol, low density lipoprotein-LDL, high density lipoprotein-HDL, triglycerides-TG) and glycemic profiles (fasting glucose, fasting insulin).

Homeostatic model assessment index (HOMAi) was calculated in order to evaluate insulin sensitivity.

The accredited Biochemistry Laboratory of the University of Rome “Tor Vergata” (Italy) performed the analyses.

### 2.2. Anthropometric Measurements

After 12 h of overnight fasting, anthropometric measurements were performed on subjects in underwear. According to standard methods, the body weight (kg) was measured to the nearest 0.01 kg, using an accurate balance scale (Invernizzi, Rome, Italy) [26].

Height (m) was measured using a stadiometry to the nearest 0.1 cm (Invernizzi, Rome, Italy). Body Mass Index (BMI) was calculated according to Quetelet Index as body weight divided by height squared (kg/m^2^) [27].

Waist circumference has been measured on the horizontal plane between the iliac crest and costal margin of the lower rib; the measure has been taken at the end of expiration. Hip circumference was measured on the horizontal plane at the great trochanter. Both measurements have beenrepeated [28].

### 2.3. Dual-Energy X-ray Absorptiometry

Lean and fat body mass were studied by DXA (iDXA, G.E. Medical Systems, Waukesha, WI, USA) [29].

The average measuring time was 20 min. The effective radiation dose from this procedure was 0.01 mSv.

This technique assesses whole and segmental body fat, lean mass and bone tissue.

The patient laid supine wearing a standard cotton t-shirt, shorts and socks. DXA scan divides the body into six compartments (head, trunk, arms, legs, android and gynoid areas). The software is able to distinguish fat and lean body mass and bone mineral content for each region.

### 2.4. Questionnaires

Two questionnaires were administered to the patients: AMS [22] and YFAS [23].

The AMS is a standardized scale according to psychometric norms, designed in 1999 in order to determine aging symptoms and their severity over the time. The questionnaire, composed of 17 questions each scoring from 1 to 5, identifies three different categories of symptoms: psychological, somato-vegetative and sexual [30]. The total AMS score may vary from 17 to 85, and distinguishes five grades of severity, ranging from no/little complaints to severe complaints.

The YFAS, on the other hand, investigates addiction symptoms related to the consumption of high fat and high sugar foods. This questionnaire is based on diagnostic criteria from the DSM IV addictive substances. Two different summary scores were used to evaluate the presence of food addiction in our population: a dichotomous diagnosis (yes/no) and a symptom count (A-H) [23]. The version of the YFAS used was “YFAS 1.0”, translated into Italian language by Innamorati et al. [31], through we evaluated changes in percentage between pre and post dietotherapy and PA treatment.

### 2.5. Nutritional Intervention

Nutritional therapy, comprising a customized nutrition plan and personalized dietary counselling, was performed based on body composition and anthropometrical features of single patient. Nutritional intervention in combination with the PA program was aimed at achieving a reduction of 10% body weight compared to baseline body weight of the subjects. The mean time of our combinatorial treatment (nutritional intervention and PA) was of 3 ± 1 months. The nutritional treatment consisted in a hypocaloric (basal metabolism estimated by De Lorenzo’s formula) [32], high-protein diet (1.5 g/kg ideal body weight/day). Diet energy gap was between 170–250 kcal/day for 10% weight loss [33]. The macronutrient composition was: carbohydrates 45–50% kcal/day; proteins 20–25% kcal/day; total fat 30% kcal/day (saturated fat < 7% kcal/day; polyunsaturated fatty acids, 10–20% kcal/day and monounsaturated fatty acids, 10–20% kcal/day; cholesterol consumption < 300 mg/day). Fiber daily intake was 25–30 g. Sodium daily intake was <5 g. No alcoholic beverages were allowed. The diet prescribed consisted in five meals following a “Mediterranean” style: breakfast, snack, lunch, afternoon snack and dinner. All macronutrients (proteins, carbohydrate, lipids) were present in each meal. The diet set was typical an Italian Mediterranean Diet, characterized by a high consumption of fruits, fresh vegetables and extra virgin olive oil. The protein source was mainly represented by vegetables (legumes and cereals) and fish [2]. The patient’s compliance was checked through nutritional counselling made by expert dietitians. All patients have been led to correct food choices. The plan for each subject was obtained from a dietetic software package (Dietosystem, DS Medica, Milan, Italy).

All subjects were advised to take probiotics as dietary regimens adjuvants.

### 2.6. Physical Activity Program

All subjects were prescribed PA, 150 min per week of aerobic activity at mild intensity (50–70% of max heart rate-HR) and/or 90 min per week activity at high intensity (>70% max HR).

All patients were recommended to practice PA at least three days per week, according to guidelines of the Italian Diabetes Society [34]. For the evaluation of compliance of prescribed PA, we performed counselling sessions. Moreover, a personal trainer who was part of our team followed the enrolled patients.

### 2.7. Statistical Analysis

All data was initially entered into an Excel spreadsheet (Microsoft, Redmond, WA, USA) and the statistical analysis was performed using the Statistical Social Package for Windows, version 15.0 (SPSS, Chicago, IL, USA). The descriptive statistics consisted of the mean ± standard deviation for parameters with normal distributions (after confirmation with histograms and the Kolgomorov-Smirnov test), the median and the interval (minimum; maximum) for variables with non-normal distributions. The comparison of the normal variables between pre and post treatment was performed with a paired T-test, also presenting the mean differences and 95% confidence interval (CI). Whilst for non-normal variables the Wilcoxon test for paired data was performed. The McNemar test was performed to compare dichotomous data before and after dietary intervention and PA. Pearson’s correlation analysis was carried out for the evaluation of a possible linear relationship between hormonal profile values and all the other variables examined.

A value of *p* < 0.05 was considered statistically significant. All graphs were produced with Excel (Microsoft, Redmond, WA, USA).

## 3. Results

Twelve (mean age 46.6 ± 14 years; range 25–63 years) out of the 14 recruited patients completed the study protocol (two of them did not reach the designated 10% weight loss).

The enrolled patients were addressed to our clinical center for the following reasons: obesity (50%), erectile dysfunction (22%), gynecomastia (21%) and couple infertility (7%).

Patients with gynecomastia were also investigated with breast ultrasound in order to exclude a true gynecomastia and to confirm pseudo-gynecomastia.

Table 1 summarizes the baseline demographic, anthropometrical and laboratory parameters of the study population.

At baseline, mean TT value 300.2 ± 79.5 ng/dL was observed, resulting in lower than the average normal value for age and sex (>349 ng/dL or 12.1 nmol/L). Mean E2 value was 46.3 ± 13.1 pg/mL, slightly higher compared to the normal range for age and sex (normal range < 45 pg/mL). Mean HOMAi value was 4.1 ± 2.3, suggesting an insulin resistance in enrolled patients. In addition, this data correlated with an android fat distribution (android/gynoid ratio 1.29 ± 0.08 as reported in Table 2), corroborating the hypothesis that visceral obesity favors insulin resistance, as demonstrated in several previous studies [35,36,37].

In this population, hypovitaminosis D (vitamin D levels below 30 ng/mL) has been shown in 93% of patients.

A significant enhancement of TT after nutritional intervention (408.3 ± 125.9 ng/dL vs. 300.2 ± 79.5 ng/dL, *p* = 0.002, 95% CI 26.8; 167.7) was observed, accompanied by a significant reduction of E2 level after a 10% weight loss (48.3 ± 14.9 pg/mL vs. 39.2 ± 15.2 pg/mL, *p* = 0.049, 95% CI 301; 0.0).

**Figure 1:**
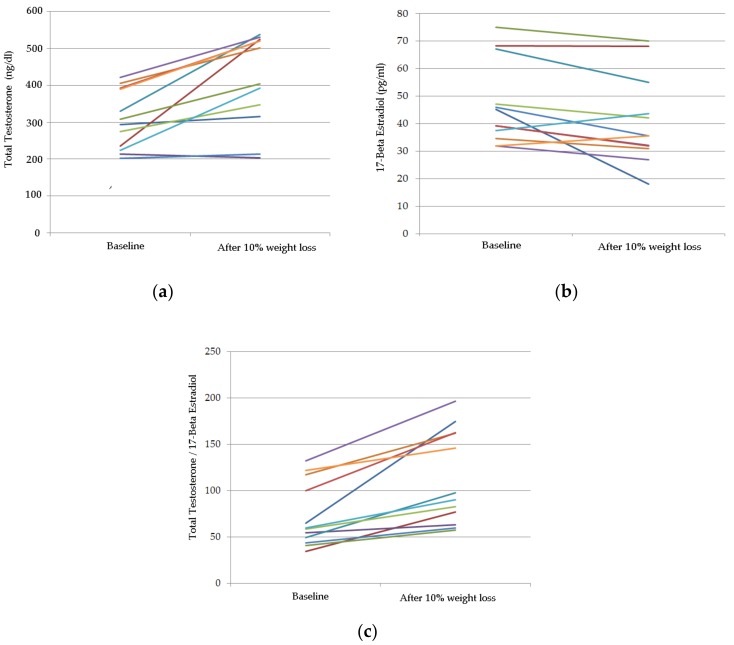
Spaghetti plot of hormonal profile at baseline and after 10% weight loss with individual patient trajectories indicated by colored lines. (**a**) TT; (**b**) E2; (**c**) aromatase enzyme activity, expressed as TT/E2.

Moreover, aromatase enzyme activity, expressed as TT/E2 ratio was significantly improved after a 10% weight loss (68.6 ± 32.6 vs. 111.5 ± 51.7, *p* = 0.003, 95% CI 10.1; 66.6) (Figure 1).

Table 2 reported body composition parameters were observed at baseline and after 10% weight loss. Total FM% was significantly decreased after nutritional intervention (39.2 ± 6.4% vs. 36.2 ± 5.8%, *p* = 0.0001, 95% CI 22.5; 62.3). Similar results were observed in android and gynoid FM% (51.5 ± 6.8% vs. 47.6 ± 6.8%, *p* = 0.001, 95% CI 0.6; 1.8; 39.2 ± 6.2% vs. 36.5 ± 6.3%, *p* = 0.0001, 95% CI 0.9; 2.0). FFM (kg) was significantly reduced at the end of the study (62.3 ± 8.2 kg vs. 60.3 ± 7.7 kg, *p* = 0.002, 95% CI 45.0; 93.0).

Vitamin D increased significantly after 10% weight loss (11.3 ± 7.4 ng/mL vs. 22.9 ± 9.9 ng/mL, *p* = 0.034, 95% CI 4.9; −25.4).

The patients following both nutritional intervention and PA prescription obtained the best outcomes. Bone mineral density was not modified during the study period.

A significant reduction in BMI was shown after 10% weight loss compared to baseline values. No significant reduction in waist-hip ratio and android/gynoid fat distribution was shown, probably as result of a weight loss, which interested all body fat districts and not only android or gynoid ones.

AMS score demonstrated that 27.2% of patients had somatovegetative symptoms and 36.4% of patients had psychological symptoms. Whilst 27.3% of patients had all three symptoms: somatovegetative, psychological, and sexual complaints. Finally, 9.1% patients had both somatovegetative and psychological symptoms.

YFAS test, as reported in Table 3, showed that at baseline 54.5% of enrolled patients were positive for food addiction versus only 9.1% was positive following a 10% weight reduction.

Moreover, we evaluated whether the single examined variables modified in relation to the entity of weight loss during the study period. An inverse, but not significant, correlation between the increase in TT levels and weight loss (*p* = 0.608; R = −0.174) was found. For all the other examined variables, no significant correlation was found between their modification during the study period and the observed weight loss. We examined other baseline predictors of changes such as age. This predictor was homogeneous in our study population, since it has been tested against weight, BMI, WHR and BMD without being statistically significant. Pearson’s correlation analysis was performed for the evaluation of the possible linear relationship between the changes observed at the end of the study in the hormonal profile and all other variables examined (such as age, baseline lipid profile, baseline anthropometric parameters, baseline questionnaire scores and vitamin D levels) but we did not find any statistical significance.

## 4. Discussion

In the present pilot study, we demonstrated that life-style changes (referred to as nutritional intervention and PA) alone were able to improve body composition, as well as hormonal and metabolic profiles. We observed an increase in TT blood levels. In accordance with this, FM%, android and gynoid FM%, and E2 blood levels were all reduced. Additionally, aromatase activity was markedly decreased after 10% weight loss. Unexpectedly, in our small sample we observed a significant reduction of FFM (kg), which is possibly correlated to the hydration status of the patients.

We hypothesize that the patients have achieved a reduction in overhydration, since nutritional intervention and PA typically induce dehydration which can result in an apparent reduction of FFM (kg) [38]. However, having only performed DXA examination to measure body composition [39] we cannot be certain, as this instrument does not quantify hydration status. A further investigation in order to assess hydration status would be required in a randomized controlled trial (RCT).

Table 2 shows that 95% CI provides the measurements of the average difference between pre and post dietoterapy and PA intervention of the body composition parameters examined; consequently, the most significant values present a wider range.

We also evaluated if the Δ weight could affect all variables examined; however, we found no significant correlation. Δ TT is inversely but not significantly correlated to Δ weight. We also examined the age of enrolled patients as a possible baseline predictor of the changes observed, against the variables examined, but it was not statistically significant.

Furthermore, we examined, through Pearson’s correlation analysis, the possible impact of all the baseline parameters on the change observed in the hormonal profile after the nutritional intervention and physical activity, finding no significant correlation.

Surprisingly, vitamin D increased significantly after the 10% weight loss. We hypothesize that its enhancement may be related to the loss of adipose tissue that stores vitamin D. A recent study demonstrated that obese subjects (OS) have more storage sites for vitamin D, suggesting that OS have a greater demand for vitamin D in deposits with a consequent reduction in their serum levels [40].

During the study, two patients dropped out from our 14-patient sample, because of their debilitating level of food addiction that prevented them from following the prescribed dietotherapy protocol.

Food addiction did not drop in a statistically significant manner after the nutritional intervention and probiotic supplementation. Regarding symptom count, we observed a reduction in the prevalence of most of them with the exception of symptoms B (persistent desire or repeated unsuccessful attempt to quit) and C (much time/activity required to obtain, use, and recover). All the observed variations are not statistically significant mainly due to the small sample.

We hypothesize that the increment of YFAS symptom B, observed after a 10% weight loss, can be ascribed to possible mood disturbances. In fact, the symptom B induces in the individual an altered mood state with a consequent and persistent anxiety, caused by food deprivation. Furthermore, we found a slight increment in C symptomatology after combined therapeutic intervention possibly due to the loss of control induced by the prescribed hypocaloric regime. However, given our small sample size, an RCT should be conducted in order to analyze the social, familial and biologic profile of each patient with conseguent greater reliability of the food addiction results.

It would be advisable, moreover, to carry out a major clinical trial in order to evaluate the action of life style changes on gut microbiota composition and the possible therapeutic response of the patients. In fact, OS show a different composition of gut microbiota which is correlated to eating behavior [41].

PA also plays a key role in the treatment of obesity secondary hypogonadism. Muscle-derived peptides, called “myokines”, released after physical activity, act on adipose tissue with anti-inflammatory effects [42]. In particular, irisin derived from the cleavage of fibronectin type III domain containing protein 5 (FNDC5) and released after exercise or muscle shivering, causes the transformation of white fat cells (storage adipose tissue) into cells with a phenotype similar to that of brown fat cells. Thus, regulating thermogenesis, exerting an anti-inflammatory action and reducing macrophage migration to adipose tissue [43,44]. Therefore, we prescribed PA in order to use fat browning as a therapeutic tool for obesity and metabolic disorders.

Other studies investigated the impact of lifestyle changes on the endocrine-metabolic profile and body composition.

Armamento-Villareal R. et al. studied the effect of lifestyle changes on hormone levels in frail obese older men. After 12 months of intervention, weight loss decreased total and free Estradiol, but showed no improvement in total and free Testosterone levels. The most important weakness of this study is that the subjects enrolled were frail, obese, older men. They are not representative of the obese men population. In this type of patients, weight loss is not the best approach for raising Testosterone levels because it can worsen age-related muscle and bone loss [45].

Another study investigated hormonal profiles after a dietary program for 8–20 weeks. They studied 24 moderately obese men and they observed that a mean weight loss of 19 kg caused the normalization of estrone, E2, TT and free Testosterone levels. However, they did not implement a PA regime and their dietary intervention consisted of a supplemented fasting program (320 kcal/day).

Compared to this study, our pilot study demonstrated that with a much milder dietary restriction (170–250 kcal/day reduction compared to basal metabolism), the same results can be achieved, thanks to the combination with PA [46].

An important meta-analysis by Corona et al. [47], addressed twenty four studies. Among these, twenty two evaluated the effects of diet or bariatric surgery and the last two investigated their combined effects on hormonal profiles in men with obesity-associated hypogonadotropic hypogonadism. The authors concluded that weight loss is associated with an increase on TT and free Testosterone levels and that bariatric surgery is more effective compared to a low-calorie diet on the hormonal profile. An important difference between our study and this meta-analysis is that we achieved a normalization of TT, E2 and aromatase levels, exclusively through lifestyle changes without having to resort to bariatric surgery.

Our pilot study has implications for human health. We show that lifestyle changes improve body composition and hormonal profile, representing a first choice therapy in the treatment of obesity secondary hypogonadism, without having to recur to Testosterone administration, avoiding cardiovascular and gastro-enteric side effects.

## 5. Conclusions

For the first time, we examined the effect of lifestyle changes in obese patients whose Testosterone levels alone did not allow the diagnosis of male hypogonadism. In fact, according to the guidelines of male hypogonadism, we selected the cut off of 12.1 nmol/L associated with the presence of one of three sexual symptoms (reduction of sexual thinking, weakness of morning erections and erectile dysfunction) to highlight borderline subjects [19,48].

Given the positive result that have been obtained, it would be optimal to further this pilot study with an RCT.

It would be ideal to perform an RCT on a large sample in order to evaluate if the lifestyle changes in subjects with sub-threshold MOSH could be a first line therapeutic strategy. Moreover, from the analysis of the possible correlations observed, it could be possible to infer which kind of patient would show a better response to this kind of intervention.

## Figures and Tables

**Table 1:** Demographic, anthropometrical and blood parameters at baseline.

**(1) Demographic and Anthropometrical Parameters:**	
Age (years)	46.6 ± 14 (min 25; max 63)
BMI (kg/m^2^)	36.2 ± 7.6 (min 26.9; max 51.5)
**(2) Blood Parameters:**	
Total Testosterone (ng/dL)	300.2 ± 79.5
17-Beta Estradiol (pg/mL)	48.3 ± 14.9
TT/E2	68.6 ± 32.6
LH (mIU/mL)	6.2 ± 1.2
SHBG (nmol/L)	21.5 ± 8.8
Prolactin (ng/mL)	11.9 ± 2.7
HOMAi	4.1 ± 2.3
25-OH vitamin D (ng/mL)	11.3 ± 7.4
ColT/HDL	4.6 ± 1.2
LDL/HDL	3.1 ± 1.1
TG/HDL	2.7 ± 0.9

Data expressed as mean ± standard deviation. The demographic and anthropometrical findings also show minimum and maximum range. BMI: Body Mass Index; TT: Total Testosterone; E2: 17-Beta Estradiol; LH: Lutein Hormone; SHBG: Sex Hormone Binding Globulin; HOMAi: Homeostatic Model Assessment Index; ColT/HDL: total cholesterol/high density lipoprotein; LDL/HDL: low density lipoprotein/high density lipoprotein; TG/HDL: triglycerides/high density lipoprotein.

**Table 2:** Body composition parameters at baseline and after 10%weight loss.

Body Composition Parameters	Baseline	After 10% Weight Loss	*p* Value	95% CI
Weight (kg)	109.3 ± 20.5	100.8 ± 19.6	0.0001	0.6; 8.0
BMI (kg/m^2^)	36.2 ± 7.6	33.4 ± 7.4	0.0001	0.5; 1.7
WHR	0.9 ± 0.1	0.9 ± 0.1	0.052	0.01; 0.00
Total FM%	39.2 ± 6.4	36.2 ± 5.8	0.0001	22.5; 62.3
Android FM%	51.5 ± 6.8	47.6 ± 6.8	0.001	0.6; 1.8
Gynoid FM%	39.2 ± 6.2	36.5 ± 6.3	0.0001	0.9; 2.0
FM L2-L5 (kg)	7.33 ± 2.7	6.0 ± 2.4	0.0001	0.4; 1.8
Total FM (kg)	42.3 ± 11.8	36.8 ± 9.9	0.0001	0.1; −0.3
Total FFM (kg)	62.3 ± 8.2	60.3 ± 7.7	0.002	45.0; 93.0
A/G	1.29 ± 0.08	1.31 ± 0.09	0.784	22.1; 86.9
BMD (g/cm^2^)	1.4 ± 0.5	1.4 ± 0.4	0.359	0.1; −0.3

Data expressed as mean ± standard deviation (SD). *p* value < 0.05 is considered significant. CI: Confidence Interval; BMI: Body Mass Index; WHR: Waist-Hip-Ratio; FM%: Fat Mass Percentage; FM: Fat Mass; FFM: Fat Free Mass; A/G: Android/Gynoid; BMD: Bone Mineral Density.

**Table 3:** Comparison of the Yale Food Addiction Scale (YFAS) results at baseline and after 10% weight loss.

	Patient Group		
	Baseline (%)	After 10% Weight Loss (%)	*p* (McNemar’s Test)
Prevalence of food addiction	54.5	9.1	0.063
**Prevalence of every symptom:**			
A. Substance taken in larger amount and for a longer period than intended	36.4	0	0.125
B. Persistent desire or repeated unsuccessful attempt to quit	36.4	54.5	0.500
C. Much time/activity required to obtain, use, and recover	18.2	27.3	0.500
D. Important social, occupational, or recreational activities given up or reduced	54.5	18.2	0.250
E. Use continues despite knowledge of adverse consequences	27.3	9.1	0.063
F. Tolerance	18.2	0	0.500
G. Withdrawal	9.1	0	1
H. Clinically significant impairment	36.4	9.1	0.250
